# Competency matrix for healthcare professionals caring for people with chronic conditions^*^

**DOI:** 10.1590/1518-8345.7751.4759

**Published:** 2026-06-15

**Authors:** Saulo Fabio Ramos, Felipa Rafaela Amadigi, Natalia Gonçalves, Roberta Waterkemper, Kenia Lara Silva, Monica Motta Lino

**Affiliations:** 1 Universidade Federal de Santa Catarina, Florianópolis, SC, Brazil.; 2 Universidade Federal de Ciências da Saúde de Porto Alegre, Departamento de Enfermagem, Porto Alegre, RS, Brazil.; 3 Escola de Enfermagem da Universidade Federal de Minas Gerais, Belo Horizonte, MG, Brazil.

**Keywords:** Competency-Based Education, Chronic Disease, Health Assessment, Nursing Education, Multidisciplinary Team, Public Health Professional Education.

## Abstract

**Objective:**

to develop a competency matrix for healthcare professionals caring for people with chronic conditions.

**Method:**

ex-post-facto impact assessment research, based on David Kolb’s Experiential Learning Theory. The recommendations of SQUIRE 2.0 were adopted. Intervention projects from the Specialization Course in Care for People with Chronic Noncommunicable Diseases were analyzed. The analysis involved pre-analysis, exploration, coding, categorization, treatment of results, inference, and interpretation using AtlasTi^®^ software.

**Results:**

337 projects were analyzed, adapted to David Kolb’s theoretical framework on the experiential learning cycle, which comprised four domains: identification, analysis, problematization, and transformation. These results supported the development of a matrix of general and specific competencies.

**Conclusion:**

the proposed matrix contributed to the qualification of health professionals, prepares specialists to work with chronic conditions and reinforced the importance of critical thinking, analytical skills, and the application of knowledge, promoting a comprehensive understanding of complex issues in learning.

## Highlights

**(1)** Competency matrix for the care of chronic conditions in public health. **(2)** Relevance of professional qualifications for dealing with chronic conditions. **(3)** Study based on David Kolb’s Theory of Experiential Learning. **(4)** Critical thinking emphasizes the importance of critical thinking in health. **(5)** The importance of analytical skills in health learning.

## Introduction

Continuing Health Education (CHE) is a strategy that integrates learning into the work process to transform health practices. Primary Health Care (PHC) has proven to be a powerful space for the consolidation of CHE, as it promotes reflection and learning based on the unique characteristics of the territory^1^. In this context, Therapeutic Patient Education (TPE) has emerged as a relevant strategy for the management of chronic noncommunicable conditions (CNCC), standing out for its role in promoting self-care and improving the management of these conditions, both at the individual and population levels^2^.

The inclusion of TPE in the training curricula of health professionals has been encouraged in several countries, especially in Europe, with measures aimed at revising workforce regulations, including specific educational programs and expanding the responsibilities of health professionals in interdisciplinary areas^2^. These actions reinforce the importance of preparing professionals not only to provide immediate care, but also to educate patients, making care more effective and comprehensive.

The competence of healthcare professionals is defined by a combination of Knowledge, Skills and Attitudes (KSA), which are essential for ensuring quality care and promoting health effectively^3^. In this sense, continuing education in health plays a central role, being a strategic tool to ensure that professionals remain up to date and trained to meet the demands of the population, especially in the context of the Unified Health System (*Sistema Único de Saúde* - SUS), which covers a vast network of workers throughout the country^4^. The 4th National Conference on Health Work and Education Management reinforced the need to value these professionals through investments in their continuing education^4^.

Among the various educational theories applicable to health training, the Experiential Learning Theory of David Kolb, an American psychologist, professor and education theorist, stands out for integrating concrete experiences and cognitive processes, promoting a learning cycle that involves active experimentation and critical reflection^5^. Experiential theory supports practices that optimize how adults construct and organize knowledge. This is done by identifying and detailing learning models and styles that are characterized by unique and predominant traits. A learning style is defined as “[...] a lasting and stable state that derives from consistent configurations of transactions between the individual and their environment”^5^.

The different learning styles are organized and defined by four adaptive models that are cyclically interconnected. In this process, the student or learner experiences Concrete Experience (CE), which corresponds to direct contact with the situation, involving doing and experiencing, based on pre-existing knowledge. This is followed by Reflective Observation (RO), which consists of an introspective movement of reflection on the action performed, aiming to identify its effects and the relationships established. Subsequently, Abstract Conceptualization (AC) occurs, characterized by the formation of abstract concepts, through the generalization of rules and principles, as well as the synthesis of the elements of the experience that were reflected upon. Finally, Active Experimentation (AE), which is an outward-looking movement, applying in practice the knowledge and thought processes that have been reflected upon, explained, and generalized^5^. 

This theory focuses on the inclusion of individual experiences, and the learning and change process occurs through the involvement of concrete emotional experiences integrated with cognitive processes^6^ ^-^ ^7^. This approach not only promotes the development of critical thinking, but also encourages students to become active participants in the process. Thus, the characteristics of experiential learning are the act of learning in practice, reflecting on knowledge, exploring the new, using theoretical concepts, and accepting challenges^8^.

In this context, the present study aimed to develop a competency matrix for health professionals caring for people with chronic conditions. The matrix was constructed based on the analysis of 337 intervention projects developed by professionals who graduated from a specialization course offered by a public university in Southern Brazil, with an emphasis on conditions such as diabetes, hypertension, obesity, and smoking, and with interventions focused on education, health promotion and care management in multidisciplinary contexts. 

## Method

This is an ex-post-facto Impact Assessment study, originating from a doctoral thesis in Nursing, which analyzed a Specialization Course in Care for People with Chronic Noncommunicable Diseases. The course was offered by a public university located in the city of Florianópolis, in Santa Catarina, in the Southern Region of Brazil and adopted Experiential Learning Theory as its theoretical and methodological framework, analyzed from this theoretical perspective. 

The writing of this study followed the guidelines of SQUIRE 2.0 (Standards for Quality Improvement Reporting Excellence), appropriate for research describing interventions aimed at improving healthcare quality. This methodological choice is justified by the nature of the research, which was based on the documentary analysis of 337 intervention projects developed by healthcare professionals during their specialization in chronic conditions. These projects, implemented in real contexts of the SUS, aimed to improve care practices, which aligns the study with the scope of SQUIRE, which seeks to promote sustained changes in the quality of care.

### Data collection and organization

The data examined for this study were obtained in 2024 and originated from the final projects of health professionals who completed the course. All 337 intervention projects (IP) were read and subjected to rigorous analysis. It is important to note that the analyzed documents, which are publicly accessible, are available on the ARES (Collection of Educational Resources in Health) online platform of UNA SUS (Open University of SUS), where the course was developed. They can be accessed at the following address: https://ares.unasus.gov.br/acervo/.

For the organization and analysis of these documents, the files were imported in Word format into Atlas.Ti^®^ software version 24.1.1.30183.

### Data analysis

Data analysis was conducted in two stages, following the operationalization of Atlas.Ti^®^ software for qualitative data, which included: Data Import; Data Organization; Coding; Code Analysis; Network Creation; Interpretation of Results; Report Generation and, finally, analysis of the networks generated by the software. Based on the concept networks created in Atlas.Ti^®^, the competency matrix was constructed, applying the theoretical and methodological model of the specialization course in the analysis process. Based on the systematization and organization of general and specific competencies, the results present textual excerpts from the final papers that illustrate the category or competency. These were characterized based on Kolb^5^ as: concrete experience (context of reality); reflective observation (problematization); abstract conceptualization (justification and feasibility) and active experimentation (method and application), as well as the process of constructing and implementing intervention projects. Each cycle was given a new concept to represent each stage of Kolb’s learning process (Figure 1) and characterize an explanatory definition of the movement performed by the graduate in this process, which were identified as domains. To improve the visualization and clarity of the information, the Miro^®^ tool was used to create the illustrative image.

**Figure 1:** Domains of the Experiential Learning Cycle of the Specialization Course Care for People with Chronic Noncommunicable Diseases. Florianópolis, SC, Brazil, 2024

**DOMAIN**	**SPECIFIC ACTIONS**	**DESCRIPTION OF THE DOMAIN**
**Concrete Experience (Identification)**	Identify, Recognize, Characterize, Diagnose, Contextualize, Describe, Map, Detect gaps	Refers to the ability to identify and contextualize the health conditions of people in the territory. It includes identifying population gaps and specific needs, facilitating individualized and effective approaches to managing chronic conditions. Practical applications include accurate diagnoses and the development of intervention strategies adapted to local conditions.
**Reflective Observation (Problem-solving)**	Examine, Observe, Perceive, Reflect, Perceive, Investigate, Measure	It encompasses the problematization of situations and contexts experienced by people. These skills are crucial for critical analysis and reflection on care practices, allowing for a more holistic and integrated approach. In practice, this translates into the ability to identify factors that hinder treatment adherence and develop strategies to overcome these challenges.
**Abstract Conceptualization (Analysis)**	Evaluate, Interpret, Analyze, Understand, Integrate, Engage, Develop	It covers the analysis and critical evaluation of health situations. These skills are fundamental to the continuous evaluation and improvement of health practices. In practical application, professionals can use these skills to conduct research, interpret data, and adjust interventions as necessary.
**Active Experimentation (Transformation)**	Implement, Innovate, Apply, Solve, Develop, Execute, Deploy, Transform	This relates to the practical application of acquired knowledge and the transformation of healthcare practices. These skills are fundamental to innovation and continuous improvement in healthcare services. In practice, this includes the ability to implement new approaches, adjust interventions as necessary, and promote a collaborative problem-solving environment within the healthcare team.

### Ethical aspects and confidentiality

Although this is a documentary study that generally does not require ethical approval, the present study obtained a favorable opinion from the Human Research Ethics Committee, under the Certificate of Presentation for Ethical Review (CAAE) number 66635322.0.0000.0121. The standards established by Resolution No. 466 of 2012, of the National Health Council and the General Data Protection Law (Lei Geral de Proteção de Dados - LGPD) were followed. 

To ensure the anonymity of the authors, the 337 Intervention Projects (IP) were coded sequentially, receiving the identification from IP 1 to IP 337. The documents were kept by a controller (a natural person responsible for decisions regarding the processing of personal data, consent of data subjects, information about their rights, and information security) and an operator (a legal entity that processes personal data under the guidance of the controller)^9^.

### Results

**Figure 2:**
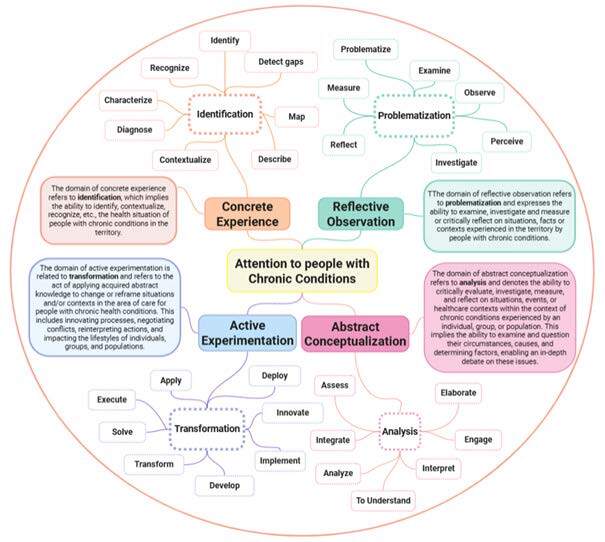
Competency matrix for dealing with chronic conditions. Florianópolis, SC, Brazil, 2024

The analysis stage, which followed the operationalization of qualitative data using ATLAS.Ti^®^ software, comprised the import, organization, and coding of data, as well as the analysis of codes. This process resulted in a network of conceptual relationships, which served as the basis for the development of the competency matrix (Figure 2), later adapted in Miro^®^ software.

In addition, a descriptive summary was prepared detailing the application of competencies in each axis of Kolb’s theory, with a special focus on the context of caring for people with chronic conditions (Figure 3). This summary comprised Axis 1: Interpersonal and interprofessional relationships and communications; Axis 2: Professionalism and Ethics; Axis 3: Focus on the person, family, and community and Axis 4: Management and evaluation.

**Figure 3:** Detailed description of the application of skills focused on the context of care for people with chronic conditions from the Specialization Course on Care for People with Chronic Noncommunicable Diseases. Florianópolis, SC, Brazil, 2024

**Axis 1: Interpersonal and interprofessional relationships and communications**
- Interact with employees and other professionals to provide integrated care to people; - Express yourself clearly and accurately, both verbally and in writing (in medical records, forms, etc.), to ensure understanding by members of the multidisciplinary team; - Adapt your speech and language to different professional categories and to the user/family; - Demonstrate teamwork skills; - Communicate ethically and effectively with all professionals and with the user/family; - Abbreviate, organize, and present clinical cases for discussion; - Collaborate and assist less experienced professionals in the learning process and in the development of tools/technologies.
**Axis 2: Professionalism and Ethics**
- Respect people’s uniqueness, individuality, and autonomy; - Observe the principle of social justice; - Demonstrate a critical, reflective, and socially responsible attitude; - Demonstrate diligence, punctuality, and proper time management; - Learn and understand the limitations of health management and therapies; - Understand and, when necessary, apply the code of ethics of your profession; - Maintain the confidentiality of information to which you have access; - Respect legal, ethical, and duty principles.
**Axis 3: Focus on the Person, Family, and Community**
- Correlate the person’s complaints with their behavior and lifestyle; - Use the principles of disease prevention and health promotion; - Consider the user’s perspective when planning care; - Assess the person’s understanding, ability to adhere to the plan, and the cost of treatment; - Adjust care plans according to individual needs; - Establish an effective therapeutic relationship with the person and their family; - Assess important life events (birth, disability, end of life) that may impact health; - Involve appropriate healthcare professionals and community resources in care; - Mobilize community services and agencies in the interests of individuals, families, and the community.
**Axis 4: Management and evaluation**
- Identify and reflect on experiences in caring for people with chronic conditions; - Analyze experiences to generate concepts and strategies that can be applied in practice; - Implement these strategies and evaluate their effectiveness, adjusting as necessary; - Use management tools to monitor and evaluate the quality of care; - Promote care processes aligned with guidelines and best practices; - Apply the best available evidence in decision-making; - Evaluate the effectiveness of decisions made and adjust practices based on results; - Identify training needs and develop training programs; - Encourage participation in professional development activities.

## Discussion

The development of a competency matrix adapted from Kolb’s theory^5^ represents an innovative and robust approach to health education, particularly focused on the management of chronic noncommunicable diseases (CNCDs). The experiential cycle, which involves the phases of concrete experience, reflective observation, abstract conceptualization, and active experimentation, offers a methodology that is highly applicable to complex and challenging clinical contexts. By following this cycle, healthcare professionals not only acquire theoretical knowledge but also improve their practical skills through critical reflection and active experimentation in real situations^6^. This integration fosters the development of skills that are indispensable for the care of chronic conditions, which require adaptive and multidisciplinary approaches^9^.

Concrete Experience, the first phase of Kolb’s cycle, aligns with Axis 1: Interpersonal and Interprofessional Relationships and Communications. At this stage, the focus is on identifying problems, recognizing gaps, and characterizing the specific healthcare needs of people with chronic conditions. This process requires active observation of real situations and continuous interaction with other professionals and patients themselves, which is essential for effective teamwork and interpersonal communication. The literature corroborates the effectiveness of communication as an important element in all health professions and institutions. It drives innovation, the development of communication strategies, and the use of technologies that promote clear and objective interaction^10^ ^-^ ^11^. In addition, the ability to adapt language to different audiences, whether the multidisciplinary team, patients, or their families, contributes significantly to increasing treatment adherence and the perception of care^11^. This is because effective communication clarifies doubts, softens feelings in the face of clinical conditions, and favors the establishment of emotional bonds, generating direct benefits for the quality of care and patient recovery^12^.

This initial phase of Concrete Experience is fundamental, as it establishes the basis for interpersonal and interprofessional engagement, promoting collaboration between different specialties. Interprofessional Primary Care (IPC), as a model of collaborative patient care, is becoming increasingly vital in the context of complex health systems and in view of the growing needs of patients^13^. Thus, Concrete Experience facilitates collaborative interactions and is seen as the starting point for creating a competency matrix focused on care for CNCC.

Reflective Observation, the second phase of the cycle, is directly linked to Axis 2: Professionalism and Ethics. At this stage, professionals engage in critical reflection on their actions, analyzing the events they have experienced and the results they have achieved. Professionals who work with chronic patients face ongoing ethical challenges, such as ensuring respect for patient autonomy and confidentiality, while promoting social justice in access to treatment. For care to reach a high ethical standard, it is essential that the principles of respect for patient autonomy and justice are properly considered. The ability to reflect on these issues allows healthcare professionals to continuously improve their practices, ensuring that their actions comply with high ethical standards.

In this phase of reflective observation, experiential learning also fosters the development of critical and creative thinking. Professionals need to be able to contextualize complex situations and deepen their reflection, a process that can be described as creative, dynamic, and transformative learning, which improves practice by promoting self-awareness and critical thinking^16^.

Thus, Axis 2, which addresses professionalism and ethics, highlights how reflective observation contributes significantly to the training of health professionals who are able to act responsibly and sensitively in the face of ethical dilemmas, especially in long-term contexts, such as CNCC.

Next, Abstract Conceptualization, the third phase of the cycle, corresponds to Axis 3: Focus on the Person, Family and Community. At this stage, healthcare professionals analyze the data collected and observations made to construct critical interpretations of patients’ health conditions. Here, the emphasis is on the ability of professionals to associate chronic conditions with social, cultural, and family determinants, in addition to providing treatment, advice on health management, and information exchange, especially for individuals living with chronic diseases^17^ ^-^ ^18^. Abstract conceptualization involves comparing current experiences with similar situations, generalizing rules and principles, and synthesizing ideas from discussions, forming a common core of shared concepts^19^. This approach allows healthcare professionals to adapt their interventions holistically, addressing the physical, emotional, and social needs of the patient.

This phase is especially relevant in the management of CNCC, where adherence to treatment and regular follow-up are essential. By integrating this critical analysis with a focus on families and communities, professionals are empowered to offer care that goes beyond medical treatment. This promotes a comprehensive approach to health, which includes patient education and the strengthening of community support networks.

Finally, Active Experimentation, the last phase of the cycle, aligns with Axis 4: Management and Evaluation. In this stage, health professionals apply the knowledge acquired directly in clinical practice. This drives the transformation of work processes and the implementation of strategies based on scientific evidence. The core of active experimentation is action: it represents the practical application of knowledge and thought processes in new situations, always with an emphasis on collaboration and teamwork^20^. 

Education about chronic diseases empowers people by providing them with the knowledge, skills, and resources necessary to better understand their condition, make effective treatment decisions, and adopt healthy lifestyle choices. This education generally covers information about the nature of chronic conditions, including their causes and symptoms, treatment options, and management strategies. In this context, continuing professional development is essential to ensure that healthcare professionals are always up to date and prepared to face the challenges posed by CNCCs^21^.

The proposed competency matrix is, therefore, an indispensable tool for aligning the theoretical principles studied with the real needs of healthcare professionals working in the management of chronic conditions. By following the phases of the Kolb cycle, professionals become capable of developing competencies that not only respond to the technical demands of care but also promote critical reflection, contextual analysis, and practical experimentation, all of which are essential for addressing the complex challenges associated with CNCC.

Additionally, the proposed competency matrix reinforces the need for Continuing Education in Health. This need is widely emphasized by the Strategic Action Plan for Addressing Chronic Diseases and Noncommunicable Diseases (*Plano DANT*), which advocates the continuous development of health professionals’ skills to deal with the growing demands of CNCDs^21^. This improvement is essential not only to enhance the quality of care provided, but also to ensure that professionals are able to act proactively and innovatively in the complex context of public health.

Thus, by integrating theory with practice through experiential learning, the competency matrix promotes an approach that prepares healthcare professionals to transform the reality of healthcare. By combining the four phases of Kolb’s cycle with the four axes of the matrix, professionals are prepared to deal with chronic conditions in an innovative, efficient, and humanized manner, contributing to the continuous improvement of health services and the well-being of the communities served.

The research highlights that most of the graduates were nurses. Thus, the competency matrix reinforces the importance of nurses as one of the pillars for cooperation in care. The skill and knowledge of these professionals in guiding educational activities with patients, families, communities, and teams, connecting them in support networks, is recognized. The practice is adapted to the local reality, enabling nurses to offer more efficient, humanized care focused on people’s real needs. This contributes significantly to improving health outcomes and implementing comprehensive care.

This study, based exclusively on qualitative data from intervention projects, has limitations inherent to its methodological design. This approach may restrict the breadth of evidence on the effectiveness of the competency matrix in different clinical practice contexts, making it difficult to generalize the findings. Furthermore, as this is an *ex-post-facto* documentary analysis, it was not possible to conduct interviews or complementary triangulations to further explore the results.

For future research, it is recommended that longitudinal studies be conducted to evaluate the application and impact of the matrix in different healthcare settings and regions. The inclusion of quantitative methodologies is also relevant for more robustly measuring the results obtained with the use of the competency matrix. Another opportunity would be to develop comparative studies between different educational approaches in order to verify the potential for replicability of experiential learning in other health and professional training contexts. Such investigations could broaden the external validity of the proposal and provide a more comprehensive understanding of its impact.

## Conclusion

The analysis of intervention projects, carried out using Kolb’s experiential learning model, revealed a list of competencies that support the management of clinical practice in the context of Chronic Health Conditions (CHC). This approach not only combines theory with practice to provide training that prepares health professionals to face the challenges of CHC in a wide variety of contexts but also broadens the scope of nursing as a leading field in health promotion.

The competency matrix developed not only prepares specialists to work with CHC, but also reinforces the importance of critical thinking, analytical skills, and the application of knowledge, promoting a greater understanding of complex issues in health learning.

## Data Availability

All data generated or analysed during this study are included in this published article.
