# Supplementary material for: Competency matrix for healthcare professionals caring for people with chronic conditions
Source: Rev Lat Am Enfermagem. 2026 Jun 15;34:e4759. doi: 10.1590/1518-8345.7751.4759 (PMC13277356; doi:10.1590/1518-8345.7751.4759)
Supplement: Translation

# Translation: Matriz de competências para profissionais de saúde no cuidado às pessoas com condições crônicas^*^

**DOI:** 10.1590/1518-8345.7751.4760

**Authors:** Saulo Fabio Ramos, Felipa Rafaela Amadigi, Natalia Gonçalves, Roberta Waterkemper, Kenia Lara Silva, Monica Motta Lino

**Affiliations:** 1 Universidade Federal de Santa Catarina, Florianópolis, SC, Brasil.; 2 Universidade Federal de Ciências da Saúde de Porto Alegre, Departamento de Enfermagem, Porto Alegre, RS, Brasil.; 3 Escola de Enfermagem da Universidade Federal de Minas Gerais, Belo Horizonte, MG, Brasil.

**Keywords:** Educação Baseada em Competências, Doença Crônica, Avaliação em Saúde, Educação em Enfermagem, Equipe Multiprofissional, Educação Profissional em Saúde Pública.

## Abstract

**Objetivo:**

desenvolver uma matriz de competências para profissionais de saúde no cuidado às pessoas com condições crônicas.

**Método:**

pesquisa de avaliação de impacto *ex-post-facto*, fundamentada na Teoria de Aprendizagem Experiencial de David Kolb. Foram adotadas as recomendações do SQUIRE 2.0. Foram analisados projetos de intervenção do Curso de Especialização em Atenção às Pessoas com Doenças Crônicas Não Transmissíveis. A análise envolveu pré-análise, exploração, codificação, categorização, tratamento dos resultados, inferência e interpretação com o *software* AtlasTi^®^.

**Resultados:**

foram analisados 337 projetos, adaptados à estrutura teórica de David Kolb sobre o ciclo de aprendizagem experiencial, que compreendeu quatro domínios: identificação, análise, problematização e transformação. Esses resultados subsidiaram a elaboração de uma matriz de competências gerais e específicas.

**Conclusão:**

a matriz proposta contribuiu para a qualificação de profissionais de saúde, preparou especialistas para trabalhar com condições crônicas e reforçou a importância do pensamento crítico, das habilidades analíticas e da aplicação do conhecimento, promovendo uma compreensão abrangente de questões complexas no aprendizado.

## Destaques

**(1)** Matriz de competências para o cuidado de condições crônicas em saúde pública. **(2)** Relevância da qualificação profissional para o enfrentamento das condições crônicas. **(3)** Estudo baseado na Teoria da Aprendizagem Experiencial de David Kolb. **(4)** Pensamento crítico enfatiza a importância do pensamento crítico em saúde. **(5)** A importância das habilidades analíticas no aprendizado em saúde.

## Introdução

A Educação Permanente em Saúde (EPS) é uma estratégia que integra a aprendizagem ao processo de trabalho para transformar as práticas em saúde. A Atenção Primária à Saúde (APS) tem se mostrado um espaço potente para a consolidação da EPS, pois promove a reflexão e a aprendizagem a partir das singularidades do território^1^. Nesse contexto, a Educação Terapêutica do Paciente (ETP) emergiu como uma estratégia relevante para o manejo de condições crônicas não transmissíveis (CCNT), destacando-se por seu papel na promoção do autocuidado e na melhoria da gestão dessas condições, tanto ao nível individual quanto populacional^2^.

A inserção da ETP nos currículos de formação de profissionais de saúde tem sido incentivada em diversos países, especialmente na Europa, com medidas voltadas à revisão das regulamentações da força de trabalho, à inclusão de programas educacionais específicos e à ampliação das responsabilidades dos profissionais de saúde em áreas interdisciplinares^2^. Essas ações reforçam a importância de preparar profissionais não apenas para prestar assistência imediata, mas também para educar os pacientes, tornando o cuidado mais eficaz e abrangente.

A competência dos profissionais de saúde é definida pela combinação de Conhecimentos, Habilidades e Atitudes (CHA), indispensáveis para garantir um atendimento de qualidade e promover a saúde de forma efetiva^3^. Nesse sentido, a educação permanente em saúde tem um papel central, sendo uma ferramenta estratégica para garantir que os profissionais mantenham-se atualizados e capacitados para atender às demandas da população, principalmente no contexto do Sistema Único de Saúde (SUS), que abrange uma vasta rede de trabalhadores em todo o país^4^. A 4ª Conferência Nacional de Gestão do Trabalho e da Educação na Saúde reforçou a necessidade de valorizar esses profissionais por meio de investimentos em sua formação contínua^4^.

Dentre as diversas teorias educacionais aplicáveis à formação em saúde, a Teoria da Aprendizagem Experiencial de David Kolb, psicólogo, professor e teórico da educação americano, destaca-se por integrar experiências concretas e processos cognitivos, promovendo um ciclo de aprendizado que envolve experimentação ativa e reflexão crítica^5^. A teoria experiencial oferece suporte para práticas que otimizam como os adultos constroem e organizam o conhecimento. Isso é feito ao identificar e detalhar modelos e estilos de aprendizagem que se caracterizam por traços únicos e predominantes. Um estilo de aprendizagem é definido como “[... ] um estado duradouro e estável que deriva de configurações consistentes das transações entre o indivíduo e o seu meio ambiente”^5^.

Os diferentes estilos de aprendizagem são organizados e definidos por quatro modelos adaptativos que se interligam de forma cíclica. Nesse processo, o estudante ou aprendiz vivencia a Experiência Concreta (EC), que corresponde ao contato direto com a situação, envolvendo o fazer e o experienciar, pautando-se em conhecimentos preexistentes. Segue-se a Observação Reflexiva (OR), que consiste em um movimento introspectivo de reflexão sobre a ação realizada, visando identificar seus efeitos e as relações estabelecidas. Posteriormente, ocorre a Conceituação Abstrata (CA), caracterizada pela formação de conceitos abstratos, por meio da generalização de regras e princípios, bem como pela síntese dos elementos da experiência que foram refletidos. Por fim, a Experimentação Ativa (EA), que é um movimento voltado para o exterior, aplicando, na prática, os conhecimentos e os processos de pensamento refletidos, explicados e generalizados^5^.

Esta teoria tem como foco a inclusão das experiências do indivíduo e o processo de aprendizagem e mudança ocorrem a partir do envolvimento de experiências emocionais concretas e integradas aos processos cognitivos^6^ ^-^ ^7^. Essa abordagem não só favorece o desenvolvimento do pensamento crítico, como também incentiva os alunos a tornarem-se participantes ativos no processo. Assim, as características da aprendizagem experiencial são o ato de aprender na prática, refletir sobre um conhecimento, explorar o novo, utilizar conceitos teóricos e aceitar desafios^8^.

Nesse contexto, o presente estudo teve como objetivo desenvolver uma matriz de competências para profissionais de saúde no cuidado às pessoas com condições crônicas. A matriz foi construída a partir da análise de 337 projetos de intervenção elaborados por profissionais egressos de um curso de especialização ofertado por uma universidade pública do Sul do Brasil, com ênfase em condições como diabetes, hipertensão, obesidade e tabagismo, e com intervenções voltadas à educação, promoção da saúde e gestão do cuidado em contextos multidisciplinares.

## Método

Trata-se de uma pesquisa de Avaliação de Impacto do tipo *ex-post-facto*, originada de uma tese de doutorado em Enfermagem, que analisou um Curso de Especialização em Atenção às Pessoas com Doenças Crônicas Não Transmissíveis. O curso foi oferecido por uma universidade pública localizada na cidade de Florianópolis, em Santa Catarina, na Região Sul do Brasil e adotou a Teoria da Aprendizagem Experiencial^5^ como seu referencial teórico-metodológico, analisado sob essa perspectiva teórica.

A redação deste estudo seguiu as diretrizes do SQUIRE 2.0 (*Standards for Quality Improvement Reporting Excellence*), adequadas para pesquisas que descrevem intervenções voltadas à qualificação do cuidado em saúde. Essa escolha metodológica justifica-se pela natureza da pesquisa, que se fundamentou na análise documental de 337 projetos de intervenção desenvolvidos por profissionais de saúde durante a especialização sobre condições crônicas. Esses projetos, implementados em contextos reais do SUS, tinham como objetivo aprimorar as práticas assistenciais, o que alinha o estudo ao escopo do SQUIRE, que busca promover mudanças sustentadas na qualidade do cuidado.

### Coleta e organização dos dados

Os dados examinados para este estudo foram obtidos em 2024 e originaram-se dos trabalhos de conclusão dos profissionais de saúde que finalizaram o curso. Todos os 337 projetos de intervenção (PI) foram lidos e submetidos a uma análise rigorosa. É importante ressaltar que os documentos analisados, de acesso público, estão disponíveis na plataforma online ARES (Acervo de Recursos Educacionais em Saúde), da UNA-SUS (Universidade Aberta do SUS), onde o curso foi desenvolvido. O acesso pode ser feito pelo seguinte endereço: https://ares.unasus.gov.br/acervo/.

Para a organização e análise desses documentos, os arquivos foram importados no formato *Word* para o *software* Atlas.Ti^®^ na versão 24.1.1.30183.

### Análise dos dados

A análise dos dados foi conduzida em duas etapas, seguindo a operacionalização do *software* Atlas.Ti^®^ para dados qualitativos, os quais incluíram: Importação de Dados; Organização dos Dados; Codificação; Análise de Códigos; Criação de Redes; Interpretação dos Resultados; Geração de Relatórios e, por fim, a análise das redes geradas pelo *software*. Com base nas redes de conceitos criadas no Atlas.Ti^®^, a matriz de competências foi construída, aplicando o modelo teórico e metodológico do curso de especialização no processo de análise. A partir da sistematização e organização das competências gerais e específicas, os resultados apresentam trechos textuais dos trabalhos de conclusão que ilustram a categoria ou competência. Essas foram caracterizadas a partir de Kolb^5^ ^)^ como: experiência concreta (contexto da realidade); observação reflexiva (problematização); conceitualização abstrata (justificativa e exequibilidade) e experimentação ativa (método e aplicação), bem como do processo de construção e implementação dos projetos de intervenção. Cada ciclo recebeu um novo conceito para representar cada etapa do processo de aprendizagem de Kolb^5^ ^)^ (Figura 1) e caracterizar uma definição elucidativa do movimento realizado pelo egresso nesse processo, os quais foram identificados como domínios^6^. Para aprimorar a visualização e clareza das informações, a ferramenta Miro^®^ foi utilizada na elaboração da imagem ilustrativa.

**Figura 1:** Domínios do Ciclo de Aprendizagem Experiencial do Curso de Especialização Atenção às Pessoas com Doenças Crônicas Não Transmissíveis. Florianópolis, SC, Brasil, 2024

DOMÍNIO	AÇÕES ESPECÍFICAS	DESCRIÇÃO DO DOMÍNIO
**Experiência Concreta (Identificação)**	Identificar, Reconhecer, Caracterizar, Diagnosticar, Contextualizar, Descrever, Mapear, Detectar lacunas	Refere-se à capacidade de identificar e contextualizar as condições de saúde das pessoas no território. Inclui identificar lacunas populacionais e necessidades específicas, facilitando abordagens individualizadas e eficazes no manejo nas condições crônicas. Aplicações práticas incluem diagnósticos precisos e desenvolvimento de estratégias de intervenção adaptadas às condições locais.
**Observação Reflexiva (Problematização)**	Problematizar, Examinar, Observar, Perceber, Refletir, Investigar, Mensurar	Engloba a problematização das situações e dos contextos vivenciados pelas pessoas. Essas competências são cruciais para a análise crítica e a reflexão sobre as práticas de cuidado, permitindo uma abordagem mais holística e integrada. Na prática, traduz-se na capacidade de identificar fatores que dificultam a adesão ao tratamento e desenvolver estratégias para superar esses desafios.
**Conceituação Abstrata (Análise)**	Avaliar, Interpretar, Analisar, Compreender, Integrar, Envolver, Elaborar	Abrange a análise e a avaliação crítica das situações de saúde. Essas competências são fundamentais para a avaliação contínua e a melhoria das práticas de saúde. Na aplicação prática, os profissionais podem utilizar essas habilidades para realizar pesquisas, interpretar dados e ajustar intervenções conforme necessário.
**Experimentação Ativa (Transformação)**	Implementar, Inovar, Aplicar, Resolver, Desenvolver, Executar, Implantar, Transformar	Relaciona-se à aplicação prática dos conhecimentos adquiridos e à transformação das práticas de saúde. Estas competências são fundamentais para a inovação e a melhoria contínua dos serviços de saúde. Na prática, inclui a capacidade de implementar novas abordagens, ajustar as intervenções conforme necessário e promover um ambiente colaborativo de resolução de problemas dentro da equipe de saúde.

### Aspectos éticos e confidencialidade

Embora se trate de uma pesquisa documental que geralmente dispensa aprovação ética, o presente estudo obteve parecer favorável do Comitê de Ética em Pesquisa com Seres Humanos, sob o Certificado de Apresentação para Apreciação Ética (CAAE) número 66635322.0.0000.0121. Foram seguidas as normas estabelecidas pela Resolução n.º 466 de 2012, do Conselho Nacional de Saúde, e a Lei Geral de Proteção de Dados (LGPD). 

Para garantir o anonimato dos autores, os 337 Projetos de Intervenção (PI) foram codificados sequencialmente, recebendo a identificação de PI 1 a PI 337. A custódia dos documentos foi realizada por um controlador (pessoa natural responsável pelas decisões sobre o tratamento de dados pessoais, consentimento dos titulares, informação sobre seus direitos e segurança das informações) e um operador (pessoa jurídica que executa o tratamento dos dados pessoais sob a orientação do controlador)^9^.

## Resultados

A etapa de análise, que seguiu a operacionalização de dados qualitativos do *software* ATLAS.Ti^®^, compreendeu a importação, organização e codificação dos dados, bem como a análise dos códigos. Esse processo resultou na rede de relações conceituais, que serviu de base para a elaboração da matriz de competências (Figura 2), adaptada posteriormente no *software* Miro^®^.

**Figura 2:** Matriz de competências para o enfrentamento das condições crônicas. Florianópolis, SC, Brasil, 2024

Adicionalmente, elaborou-se uma síntese descritiva que pormenoriza a aplicação das competências em cada eixo da teoria de Kolb, com especial enfoque no contexto do cuidado às pessoas com condições crônicas (Figura 3). Essa síntese compreendeu o Eixo 1: Relações e comunicações interpessoais e interprofissionais; o Eixo 2: Profissionalismo e Ética; o Eixo 3: Enfoque dirigido à pessoa, família e comunidade e o Eixo 4: Gestão e avaliação.

**Figura 3:** Detalhamento da aplicação das competências enfocadas no contexto de cuidado às pessoas com condições crônicas do Curso de Especialização Atenção às Pessoas com Doenças Crônicas Não Transmissíveis. Florianópolis, SC, Brasil, 2024

**Eixo 1: Relações e comunicações interpessoais e interprofissionais**
- Interagir com os colaboradores e demais profissionais para um atendimento integrado às pessoas; - Expressar-se de forma clara e precisa, verbalmente e por escrito (em prontuários, formulários, entre outros), para garantir a compreensão dos membros da equipe multiprofissional; - Adaptar sua fala e linguagem às diferentes categorias profissionais e ao usuário/família; - Demonstrar habilidades de trabalho em equipe; - Comunicar-se de forma ética e eficaz com todos os profissionais e com o usuário/família; - Abreviar, organizar e apresentar casos clínicos para discussão; - Colaborar e auxiliar os profissionais menos experientes no processo de aprendizagem e no desenvolvimento de ferramentas/tecnologias.
**Eixo 2: Profissionalismo e Ética**
- Respeitar a singularidade, individualidade e autonomia das pessoas; - Observar o princípio da justiça social; - Demonstrar postura crítica, reflexiva e de responsabilidade social; - Demonstrar assiduidade, pontualidade e gestão adequada do tempo; - Aprender e compreender as limitações dos manejos e terapias de saúde; - Entender e, quando necessário, aplicar o código de ética da sua categoria; - Manter a confidencialidade das informações às quais se tem acesso; - Respeitar os princípios legais, éticos e de dever.
**Eixo 3: Enfoque dirigido na Pessoa, Família e Comunidade**
- Correlacionar as queixas da pessoa com seu comportamento e estilo de vida; - Utilizar os princípios de prevenção de agravos e promoção da saúde; - Considerar a perspectiva do usuário no planejamento do cuidado; - Avaliar a compreensão da pessoa, a capacidade de adesão ao plano e o custo do tratamento; - Ajustar os planos de cuidados conforme as necessidades individuais; - Estabelecer uma relação terapêutica eficaz com a pessoa e sua família; - Avaliar eventos importantes da vida (nascimento, incapacidade, fim da vida) que possam impactar a saúde; - Envolver profissionais de saúde e recursos comunitários apropriados aos cuidados; - Mobilizar serviços e agências comunitárias em prol dos interesses das pessoas, famílias e da comunidade.
**Eixo 4: Gestão e avaliação**
- Identificar e refletir sobre experiências no cuidado às pessoas com condições crônicas; - Analisar as experiências para gerar conceitos e estratégias que possam ser aplicados na prática; - Implementar essas estratégias e avaliar sua eficácia, ajustando conforme necessário; - Utilizar ferramentas de gestão para monitorar e avaliar a qualidade do cuidado; - Promover processos de cuidado alinhados com as diretrizes e as melhores práticas; - Aplicar as melhores evidências disponíveis na tomada de decisões; - Avaliar a eficácia das decisões tomadas e ajustar as práticas com base nos resultados; - Identificar necessidades de formação e desenvolver programas de capacitação; - Incentivar a participação em atividades de desenvolvimento profissional.

## Discussão

O desenvolvimento de uma matriz de competências adaptada da teoria de Kolb^5^ ^)^ representa uma abordagem inovadora e robusta para o ensino em saúde, particularmente voltada para o manejo das CCNT. O ciclo experiencial que envolve as fases de experiência concreta, observação reflexiva, conceitualização abstrata e experimentação ativa, oferece uma metodologia altamente aplicável aos contextos clínicos complexos e desafiadores. Ao seguir esse ciclo, os profissionais de saúde não apenas adquirem conhecimento teórico, mas também aprimoram suas habilidades práticas por meio da reflexão crítica e da experimentação ativa em situações reais^6^. Essa integração fomenta o desenvolvimento de competências indispensáveis para o cuidado de condições crônicas, que exigem abordagens adaptativas e multidisciplinares^9^. 

A Experiência Concreta, a primeira fase do ciclo de Kolb, alinha-se com o Eixo 1: Relações e Comunicações Interpessoais e Interprofissionais. Nesta etapa, o foco está na identificação de problemas, no reconhecimento de lacunas e na caracterização das necessidades específicas do cuidado em saúde para pessoas com condições crônicas. Esse processo exige a observação ativa de situações reais e a interação contínua com outros profissionais e com os próprios pacientes, o que é fundamental para o trabalho em equipe eficaz e a comunicação interpessoal. A literatura corrobora a eficácia da comunicação como um elemento importante em todas as profissões de saúde e nas instituições. Ela impulsiona a inovação, o desenvolvimento de estratégias de comunicação e o uso de tecnologias que promovam uma interação clara e objetiva^10^ ^-^ ^11^. Além disso, a capacidade de adaptar a linguagem para diferentes públicos, seja a equipe multiprofissional, sejam os pacientes e suas famílias, contribui significativamente para aumentar a adesão ao tratamento e a percepção de cuidado^11^. Isso ocorre porque uma comunicação eficaz esclarece dúvidas, suaviza sentimentos diante das condições clínicas e favorece o estabelecimento de vínculo afetivo, gerando benefícios diretos para a qualidade da assistência e para a recuperação dos pacientes^12^.

Essa fase inicial da Experiência Concreta é fundamental, pois estabelece a base para o engajamento interpessoal e interprofissional, promovendo a colaboração entre diferentes especialidades. A Atenção Primária Interprofissional (*Interprofessional Primary Care* - IPC), como um modelo de assistência colaborativa ao paciente, torna-se cada vez mais vital no cenário de sistemas de saúde complexos e diante das crescentes necessidades dos pacientes^13^. Dessa forma, a Experiência Concreta facilita as interações colaborativas e é vista como o ponto de partida para a criação de uma matriz de competências focada na atenção às CCNT.

A Observação Reflexiva, segunda fase do ciclo, está diretamente ligada ao Eixo 2: Profissionalismo e Ética. Nessa etapa, o profissional dedica-se a refletir criticamente sobre as ações realizadas, analisando os eventos vivenciados e os resultados obtidos. Profissionais que trabalham com pacientes crônicos enfrentam desafios éticos contínuos, como garantir o respeito à autonomia dos pacientes e à confidencialidade, ao mesmo tempo que promovem a justiça social no acesso aos tratamentos^14^. Para que a assistência alcance um patamar de alta qualidade ética, é imprescindível que os princípios de respeito à autonomia do paciente e justiça sejam devidamente considerados^15^. A capacidade de refletir sobre essas questões permite que os profissionais de saúde aprimorem continuamente suas práticas, assegurando que suas ações obedeçam a padrões éticos elevados.

Nessa fase de observação reflexiva, a aprendizagem experiencial também fomenta o desenvolvimento de uma visão crítica e criativa. Profissionais precisam ser capazes de contextualizar situações complexas e aprofundar a reflexão, um processo que pode ser descrito como um aprendizado criativo, dinâmico e transformador, que aprimora a prática ao promover a autoconsciência e o pensamento crítico^16^.

Dessa forma, o Eixo 2, que aborda o profissionalismo e a ética, evidencia como a observação reflexiva contribui significativamente para a formação de profissionais de saúde aptos a atuar com responsabilidade e sensibilidade diante dos dilemas éticos, especialmente em contextos de longa duração, como as CCNT.

Na sequência, a Conceitualização Abstrata, a terceira fase do ciclo, corresponde ao Eixo 3: Enfoque na Pessoa, Família e Comunidade. Nessa etapa, os profissionais de saúde analisam os dados coletados e as observações realizadas para construir interpretações críticas sobre as condições de saúde dos pacientes. Aqui, a ênfase recai na capacidade dos profissionais de associar as condições crônicas aos determinantes sociais, culturais e familiares, além de prover tratamento, aconselhamento sobre gestão da saúde e troca de informações, especialmente para indivíduos que convivem com doenças crônicas^17^ ^-^ ^18^. A conceitualização abstrata envolve comparar a experiência atual com situações semelhantes, generalizar regras e princípios, e sintetizar ideias a partir de discussões, formando um núcleo comum de conceitos compartilhados^19^. Essa abordagem permite que os profissionais de saúde adaptem suas intervenções de forma holística, atendendo às necessidades físicas, emocionais e sociais do paciente.

Essa fase é especialmente relevante no manejo das CCNT, em que a adesão ao tratamento e o acompanhamento regular são fundamentais. Ao integrar essa análise crítica com o foco nas famílias e comunidades, os profissionais são capacitados a oferecer um cuidado que vai além do tratamento médico. Isso promove uma abordagem integral da saúde, que inclui a educação do paciente e o fortalecimento de redes de apoio comunitários.

Por fim, a Experimentação Ativa, última fase do ciclo, alinha-se ao Eixo 4: Gestão e Avaliação. Nessa etapa, os profissionais de saúde aplicam o conhecimento adquirido diretamente na prática clínica. Isso impulsiona a transformação dos processos de trabalho e a implementação de estratégias baseadas em evidências científicas. O cerne da experimentação ativa é a ação: ela representa a aplicação prática de conhecimentos e processos de pensamento em novas situações, sempre com ênfase na colaboração e no trabalho em equipe^20^. 

A educação sobre doenças crônicas capacita as pessoas, fornecendo-lhes o conhecimento, a competência e os recursos necessários para compreender melhor sua condição, tomar decisões eficazes sobre o tratamento e adotar escolhas de estilo de vida saudáveis. Essa educação geralmente abrange informações sobre a natureza das condições crônicas, incluindo suas causas e sintomas, opções de tratamento e estratégias de gestão. Nesse contexto, o desenvolvimento profissional contínuo é essencial para garantir que os profissionais de saúde estejam sempre atualizados e preparados para enfrentar os desafios impostos pelas CCNT^21^.

A matriz de competências proposta é, portanto, uma ferramenta indispensável para alinhar os princípios teóricos estudados com as necessidades reais dos profissionais de saúde que atuam no manejo de condições crônicas. Ao se seguir as fases do ciclo de Kolb, os profissionais tornam-se capacitados a desenvolver competências que não apenas respondem às demandas técnicas do cuidado, mas também promovem a reflexão crítica, a análise contextual e a experimentação prática, todas essenciais para o enfrentamento dos desafios complexos associados às CCNT.

Adicionalmente, a matriz de competências proposta reforça a necessidade de uma Educação Permanente em Saúde. Essa necessidade é amplamente enfatizada pelo Plano de Ações Estratégicas para o Enfrentamento das Doenças Crônicas e Agravos Não Transmissíveis (Plano DANT), que preconiza o desenvolvimento contínuo das habilidades dos profissionais de saúde para lidar com as crescentes demandas das Doenças Crônicas Não Transmissíveis (DCNT)^21^. Esse aprimoramento é essencial não apenas para melhorar a qualidade do cuidado prestado, mas também para garantir que os profissionais estejam aptos a atuar de maneira proativa e inovadora no complexo contexto da saúde pública.

Assim, a matriz de competências, ao integrar a teoria com a prática por meio da aprendizagem experiencial, promove uma abordagem que prepara os profissionais de saúde para transformar a realidade do cuidado em saúde. Ao unir as quatro fases do ciclo de Kolb com os quatro eixos da matriz, os profissionais são preparados para enfrentar as condições crônicas de maneira inovadora, eficiente e humanizada, contribuindo para a melhoria contínua dos serviços de saúde e para o bem-estar das comunidades atendidas.

A pesquisa salienta que a maioria dos egressos era composta por enfermeiros. Desse modo, a matriz de competências reforça a importância do enfermeiro como um dos pilares para a cooperação no atendimento. Reconhece-se a habilidade e o conhecimento desses profissionais em orientar atividades educativas junto a pacientes, família, comunidade e equipes, conectando-os em redes de apoio. A prática ajusta-se à realidade local, possibilitando ao enfermeiro oferecer um cuidado mais eficiente, humanizado e focado nas verdadeiras necessidades das pessoas. Isso contribui de forma significativa para a melhoria dos resultados de saúde e para a efetivação da atenção integral.

Este estudo, por basear-se exclusivamente em dados qualitativos oriundos de projetos de intervenção, apresenta limitações inerentes ao seu desenho metodológico. Essa abordagem pode restringir a amplitude das evidências sobre a eficácia da matriz de competências em diferentes contextos de prática clínica, dificultando a generalização dos achados. Além disso, por se tratar de uma análise documental *ex-post-facto*, não foi possível realizar entrevistas ou triangulações complementares para aprofundar os resultados.

Para pesquisas futuras, recomenda-se a realização de estudos longitudinais que possam avaliar a aplicação e o impacto da matriz em diversos cenários de saúde e regiões. A inclusão de metodologias quantitativas também se mostra relevante para mensurar de forma mais robusta os resultados obtidos com o uso da matriz de competências. Outra oportunidade seria o desenvolvimento de estudos comparativos entre diferentes abordagens educacionais, a fim de verificar o potencial de replicabilidade da aprendizagem experiencial em outros contextos de saúde e formação profissional. Tais investigações poderiam ampliar a validade externa da proposta e fornecer uma compreensão mais abrangente de seu impacto.

## Conclusão

A análise dos projetos de intervenção, realizada por meio do modelo de aprendizagem experiencial de Kolb, permitiu desvelar um rol de competências que apoiam a gestão da prática clínica no âmbito das Condições Crônicas de Saúde (CCS). Essa abordagem não apenas combina a teoria com a prática para proporcionar uma formação que prepare os profissionais de saúde para enfrentarem os desafios das CCS nos mais variados contextos, mas também amplia o escopo de atuação da Enfermagem como campo protagonista na promoção da saúde.

A matriz de competências desenvolvida, além de preparar especialistas para atuar com CCS, reforça a importância do pensamento crítico, das características analíticas e da aplicação do conhecimento, promovendo uma maior compreensão de questões complexas no aprendizado em saúde.

## Data Availability

Todos os dados gerados ou analisados durante este estudo estão incluídos neste artigo publicado.

# Translation: Matriz de competencias para profesionales de la salud en la atención a personas con condiciones crónicas^*^

**DOI:** 10.1590/1518-8345.7751.4758

**Authors:** Saulo Fabio Ramos, Felipa Rafaela Amadigi, Natalia Gonçalves, Roberta Waterkemper, Kenia Lara Silva, Monica Motta Lino

**Affiliations:** 1 Universidade Federal de Santa Catarina, Florianópolis, SC, Brasil.; 2 Universidade Federal de Ciências da Saúde de Porto Alegre, Departamento de Enfermagem, Porto Alegre, RS, Brasil.; 3 Escola de Enfermagem da Universidade Federal de Minas Gerais, Belo Horizonte, MG, Brasil.

**Keywords:** Educación Basada em Competencias, Enfermedad Crónica, Evaluación em Salud, Educación em Enfermería, Equipo Multiprofesional, Educación em Salud Pública Profesional

## Abstract

**Objetivo:**

desarrollar una matriz de competencias para profesionales de la salud en la atención a personas con condiciones crónicas.

**Método:**

investigación de evaluación de impacto *ex post facto*, fundamentada en la Teoría del Aprendizaje Experiencial de David Kolb. Se adoptaron las recomendaciones del SQUIRE 2.0. Se analizaron proyectos de intervención del Curso de Especialización en Atención a las Personas con Enfermedades Crónicas no Transmisibles. El análisis involucró preanálisis, exploración, codificación, categorización, tratamiento de los resultados, inferencia e interpretación con el *software* AtlasTi^®^.

**Resultados:**

se analizaron 337 proyectos, adaptados al marco teórico de David Kolb sobre el ciclo de aprendizaje experiencial, que comprendió cuatro dominios: identificación, análisis, problematización y transformación. Estos resultados respaldaron la elaboración de una matriz de competencias generales y específicas.

**Conclusión:**

la matriz propuesta contribuye a la cualificación de profesionales de la salud, preparó a especialistas para trabajar con condiciones crónicas y reforzó la importancia del pensamiento crítico, de las habilidades analíticas y de la aplicación del conocimiento, promoviendo una comprensión amplia de cuestiones complejas en el aprendizaje.

## Destacados

**(1)** Matriz de competencias para la atención de condiciones crónicas en salud pública. **(2)** Relevancia de la cualificación profesional para el abordaje de las condiciones crónicas. **(3)** Estudio basado en la Teoría del Aprendizaje Experiencial de David Kolb. **(4)** Pensamiento crítico: enfatiza la importancia del pensamiento crítico en salud. **(5)** La importancia de las habilidades analíticas en el aprendizaje en salud.

## Introducción

La Educación Permanente en Salud (EPS) es una estrategia que integra el aprendizaje al proceso de trabajo para transformar las prácticas en salud. La Atención Primaria de Salud (APS) se ha mostrado un espacio potente para la consolidación de la EPS, pues promueve la reflexión y el aprendizaje a partir de las singularidades del territorio^1^. En este contexto, la Educación Terapéutica del Paciente (ETP) emergió como una estrategia relevante para el manejo de condiciones crónicas no transmisibles (CCNT), destacándose por su papel en la promoción del autocuidado y en la mejora de la gestión de esas condiciones, tanto a nivel individual como poblacional^2^.

La incorporación de la ETP en los planes de estudio de formación de profesionales de la salud ha sido incentivada en diversos países, especialmente en Europa, con medidas orientadas a la revisión de las normativas de la fuerza de trabajo, a la inclusión de programas educativos específicos y a la ampliación de las responsabilidades de los profesionales de la salud en áreas interdisciplinares^2^. Estas acciones refuerzan la importancia de preparar profesionales no solo para prestar asistencia inmediata, sino también para educar a los pacientes, haciendo el cuidado más eficaz e integral.

La competencia de los profesionales de la salud se define por la combinación de Conocimientos, Habilidades y Actitudes (CHA), indispensables para garantizar una atención de calidad y promover la salud de forma efectiva^3^. En este sentido, la educación permanente en salud desempeña un papel central, siendo una herramienta estratégica para asegurar que los profesionales se mantengan actualizados y capacitados para atender las demandas de la población, principalmente en el contexto del Sistema Único de Salud (SUS), que abarca una amplia red de trabajadores en todo el país^4^. La 4ª Conferencia Nacional de Gestión del Trabajo y de la Educación en Salud reforzó la necesidad de valorizar a esos profesionales mediante inversiones en su formación continua^4^.

Entre las diversas teorías educacionales aplicables a la formación en salud, la Teoría del Aprendizaje Experiencial de David Kolb, psicólogo, profesor y teórico de la educación estadounidense, se destaca por integrar experiencias concretas y procesos cognitivos, promoviendo un ciclo de aprendizaje que involucra experimentación activa y reflexión crítica^5^. La teoría experiencial ofrece soporte para prácticas que optimizan cómo las personas adultas construyen y organizan el conocimiento. Ello se hace al identificar y detallar modelos y estilos de aprendizaje que se caracterizan por rasgos únicos y predominantes. Un estilo de aprendizaje se define como “[...] un estado duradero y estable que deriva de configuraciones consistentes de las transacciones entre el individuo y su entorno”^5^.

Los diferentes estilos de aprendizaje se organizan y definen por cuatro modelos adaptativos que se interconectan de forma cíclica. En ese proceso, el estudiante o aprendiz vivencia la Experiencia Concreta (EC), que corresponde al contacto directo con la situación, implicando el hacer y el experienciar, basándose en conocimientos preexistentes. Le sigue la Observación Reflexiva (OR), que consiste en un movimiento introspectivo de reflexión sobre la acción realizada, con el objetivo de identificar sus efectos y las relaciones establecidas. Posteriormente, ocurre la Conceptualización Abstracta (CA), caracterizada por la formación de conceptos abstractos, por medio de la generalización de reglas y principios, así como por la síntesis de los elementos de la experiencia que fueron objeto de reflexión. Por último, la Experimentación Activa (EA), que es un movimiento orientado hacia el exterior, aplicando en la práctica los conocimientos y procesos de pensamiento reflexionados, explicados y generalizados^5^.

Esta teoría tiene como foco la inclusión de las experiencias del individuo, y el proceso de aprendizaje y cambio ocurre a partir del involucramiento de experiencias emocionales concretas e integradas a los procesos cognitivos^6^ ^-^ ^7^. Este enfoque no solo favorece el desarrollo del pensamiento crítico, sino que también incentiva a los estudiantes a convertirse en participantes activos en el proceso. Así, las características del aprendizaje experiencial son el acto de aprender en la práctica, reflexionar sobre un conocimiento, explorar lo nuevo, utilizar conceptos teóricos y aceptar desafíos^8^.

En este contexto, el presente estudio tuvo como objetivo desarrollar una matriz de competencias para profesionales de la salud en la atención a personas con condiciones crónicas. La matriz fue construida a partir del análisis de 337 proyectos de intervención elaborados por profesionales egresados de un curso de especialización ofrecido por una universidad pública del Sur de Brasil, con énfasis en condiciones como diabetes, hipertensión, obesidad y tabaquismo, y con intervenciones orientadas a la educación, la promoción de la salud y la gestión del cuidado en contextos multidisciplinarios.

## Método

Se trata de una investigación de Evaluación de Impacto de tipo *ex post facto*, originada de una tesis doctoral en Enfermería, que analizó un Curso de Especialización en Atención a las Personas con Enfermedades Crónicas no Transmisibles. El curso fue ofrecido por una universidad pública ubicada en la ciudad de Florianópolis, en Santa Catarina, en la Región Sur de Brasil y adoptó la Teoría del Aprendizaje Experiencial^5^ como su referencial teórico-metodológico, siendo analizado bajo esa perspectiva teórica. 

La redacción de este estudio siguió las directrices de SQUIRE 2.0 (*Standards for Quality Improvement Reporting Excellence*), adecuadas para investigaciones que describen intervenciones orientadas a la cualificación del cuidado en salud. Esta elección metodológica se justifica por la naturaleza de la investigación, que se fundamentó en el análisis documental de 337 proyectos de intervención desarrollados por profesionales de la salud durante la especialización sobre condiciones crónicas. Esos proyectos, implementados en contextos reales del SUS, tenían como objetivo mejorar las prácticas asistenciales, lo que alinea el estudio con el alcance de SQUIRE, que busca promover cambios sostenidos en la calidad de la atención.

### Recolección y organización de los datos

Los datos examinados para este estudio se obtuvieron en 2024 y se originaron de los trabajos de conclusión de los profesionales de la salud que finalizaron el curso. Los 337 proyectos de intervención (PI) fueron leídos y sometidos a un análisis riguroso. Es importante resaltar que los documentos analizados, de acceso público, están disponibles en la plataforma online ARES (Acervo de Recursos Educacionales en Salud), de la UNA-SUS (*Universidade Aberta do SUS*), donde se desarrolló el curso. El acceso puede realizarse mediante la siguiente dirección: https://ares.unasus.gov.br/acervo/.

Para la organización y el análisis de esos documentos, los archivos fueron importados en formato *Word* al *software* Atlas.Ti^®^ en la versión 24.1.1.30183.

### Análisis de los datos

El análisis de los datos se condujo en dos etapas, siguiendo la operacionalización del *software* Atlas.Ti^®^ para datos cualitativos, que incluyeron: Importación de datos; Organización de los datos; Codificación; Análisis de códigos; Creación de redes; Interpretación de los resultados; Generación de informes y, por último, el análisis de las redes generadas por el *software*. Con base en las redes de conceptos creadas en Atlas.Ti^®^, se construyó la matriz de competencias, aplicando el modelo teórico y metodológico del curso de especialización en el proceso de análisis. A partir de la sistematización y organización de las competencias generales y específicas, los resultados presentan fragmentos textuales de los trabajos de conclusión que ilustran la categoría o competencia. Estas fueron caracterizadas a partir de Kolb^5^ como: experiencia concreta (contexto de la realidad); observación reflexiva (problematización); conceptualización abstracta (justificación y viabilidad) y experimentación activa (método y aplicación), así como el proceso de construcción e implementación de los proyectos de intervención. Cada ciclo recibió un nuevo concepto para representar cada etapa del proceso de aprendizaje de Kolb^5^ ^)^ (Figura 1) y caracterizar una definición aclaratoria del movimiento realizado por el egresado en ese proceso, los cuales fueron identificados como dominios^6^. Para mejorar la visualización y la claridad de la información, se utilizó la herramienta Miro^®^ en la elaboración de la imagen ilustrativa.

**Figura 1:** Dominios del Ciclo de Aprendizaje Experiencial del Curso de Especialización Atención a las Personas con Enfermedades Crónicas No Transmisibles. Florianópolis, SC, Brasil, 2024

**DOMINIO**	**ACCIONES ESPECÍFICAS**	**DESCRIPCIÓN DEL DOMINIO**
**Experiencia Concreta (Identificación)**	Identificar, Reconocer, Caracterizar, Diagnosticar, Contextualizar, Describir, Mapear, Detectar brechas	Se refiere a la capacidad de identificar y contextualizar las condiciones de salud de las personas en el territorio. Incluye identificar brechas poblacionales y necesidades específicas, facilitando abordajes individualizados y eficaces en el manejo de las condiciones crónicas. Las aplicaciones prácticas incluyen diagnósticos precisos y el desarrollo de estrategias de intervención adaptadas a las condiciones locales.
**Observación Reflexiva (Problematización)**	Problematizar, Examinar, Observar, Percibir, Reflexionar, Investigar, Medir	Abarca la problematización de las situaciones y los contextos vivenciados por las personas. Estas competencias son cruciales para el análisis crítico y la reflexión sobre las prácticas de cuidado, permitiendo un abordaje más holístico e integrado. En la práctica, se traduce en la capacidad de identificar factores que dificultan la adherencia al tratamiento y desarrollar estrategias para superar esos desafíos.
**Conceptualización Abstracta (Análisis)**	Evaluar, Interpretar, Analizar, Comprender, Integrar, Involucrar, Elaborar	Abarca el análisis y la evaluación crítica de las situaciones de salud. Estas competencias son fundamentales para la evaluación continua y la mejora de las prácticas de salud. En la aplicación práctica, los profesionales pueden utilizar estas habilidades para realizar investigaciones, interpretar datos y ajustar intervenciones según sea necesario.
**Experimentación Activa (Transformación)**	Implementar, Innovar, Aplicar, Resolver, Desarrollar, Ejecutar, Implantar, Transformar	Se relaciona con la aplicación práctica de los conocimientos adquiridos y con la transformación de las prácticas de salud. Estas competencias son fundamentales para la innovación y la mejora continua de los servicios de salud. En la práctica, incluye la capacidad de implementar nuevos abordajes, ajustar las intervenciones según sea necesario y promover un entorno colaborativo de resolución de problemas dentro del equipo de salud.

### Aspectos éticos y confidencialidad

Aunque se trata de una investigación documental que generalmente dispensa aprobación ética, el presente estudio obtuvo dictamen favorable del Comité de Ética en Investigación con Seres Humanos, bajo el Certificado de Presentación para Apreciación Ética (CAAE) número 66635322.0.0000.0121. Se siguieron las normas establecidas por la Resolución nº 466 de 2012, del Consejo Nacional de Salud, y la Ley General de Protección de Datos (LGPD).

Para garantizar el anonimato de los autores, los 337 Proyectos de Intervención (PI) fueron codificados secuencialmente, recibiendo la identificación de PI 1 a PI 337. La custodia de los documentos fue realizada por un controlador (persona natural responsable de las decisiones sobre el tratamiento de datos personales, el consentimiento de los titulares, la información sobre sus derechos y la seguridad de la información) y un operador (persona jurídica que ejecuta el tratamiento de los datos personales bajo la orientación del controlador)^9^.

## Resultados

La etapa de análisis, que siguió la operacionalización de datos cualitativos del *software* ATLAS.Ti^®^, comprendió la importación, organización y codificación de los datos, así como el análisis de los códigos. Este proceso dio lugar a la red de relaciones conceptuales, que sirvió de base para la elaboración de la matriz de competencias (Figura 2), posteriormente adaptada en el *software* Miro^®^.

**Figura 2:** Matriz de competencias para el abordaje de las condiciones crónicas. Florianópolis, SC, Brasil, 2024

Adicionalmente, se elaboró una síntesis descriptiva que detalla la aplicación de las competencias en cada eje de la teoría de Kolb, con especial enfoque en el contexto del cuidado a las personas con condiciones crónicas (Figura 3). Esta síntesis comprendió el Eje 1: Relaciones y comunicaciones interpersonales e interprofesionales; el Eje 2: Profesionalismo y Ética; el Eje 3: Enfoque dirigido a la persona, la familia y la comunidad y el Eje 4: Gestión y evaluación.

**Figura 3:** Pormenorización de la aplicación de las competencias enfocadas en el contexto de cuidado a las personas con condiciones crónicas del Curso de Especialización Atención a las Personas con Enfermedades Crónicas no Transmisibles. Florianópolis, SC, Brasil, 2024

**Relaciones y comunicaciones interpersonales e interprofesionales**
- Interactuar con los colaboradores y demás profesionales para una atención integrada a las personas; - Expresarse de forma clara y precisa, verbalmente y por escrito (en historias clínicas, formularios, entre otros), para garantizar la comprensión de los miembros del equipo multiprofesional; - Adaptar su habla y lenguaje a las diferentes categorías profesionales y al usuario/familia; - Demostrar habilidades de trabajo en equipo; - Comunicarse de forma ética y eficaz con todos los profesionales y con el usuario/familia; - Abreviar, organizar y presentar casos clínicos para discusión; - Colaborar y auxiliar a los profesionales menos experimentados en el proceso de aprendizaje y en el desarrollo de herramientas/tecnologías.
**Eje 2: Profesionalismo y Ética**
- Respetar la singularidad, individualidad y autonomía de las personas; - Observar el principio de justicia social; - Demostrar postura crítica, reflexiva y de responsabilidad social; - Demostrar asiduidad, puntualidad y gestión adecuada del tiempo; - Aprender y comprender las limitaciones de los manejos y terapias de salud; - Entender y, cuando sea necesario, aplicar el código de ética de su categoría profesional; - Mantener la confidencialidad de la información a la que se tiene acceso; - Respetar los principios legales, éticos y de deber.
**Eje 3: Enfoque dirigido a la Persona, la Familia y la Comunidad**
- Correlacionar las quejas de la persona con su comportamiento y estilo de vida; - Utilizar los principios de prevención de daños y promoción de la salud; - Considerar la perspectiva del usuario en la planificación del cuidado; - Evaluar la comprensión de la persona, la capacidad de adherencia al plan y el costo del tratamiento; - Ajustar los planes de cuidados conforme a las necesidades individuales; - Establecer una relación terapéutica eficaz con la persona y su familia; - Evaluar acontecimientos importantes de la vida (nacimiento, incapacidad, fin de la vida) que puedan impactar la salud; - Involucrar a profesionales de la salud y recursos comunitarios apropiados en los cuidados; - Movilizar servicios y agencias comunitarias en favor de los intereses de las personas, las familias y la comunidad.
**Eje 4: Gestión y evaluación**
- Identificar y reflexionar sobre experiencias en el cuidado a las personas con condiciones crónicas; - Analizar las experiencias para generar conceptos y estrategias que puedan aplicarse en la práctica; - Implementar esas estrategias y evaluar su eficacia, ajustando cuando sea necesario; - Utilizar herramientas de gestión para monitorear y evaluar la calidad del cuidado; - Promover procesos de cuidado alineados con las directrices y las mejores prácticas; - Aplicar la mejor evidencia disponible en la toma de decisiones; - Evaluar la eficacia de las decisiones tomadas y ajustar las prácticas con base en los resultados; - Identificar necesidades de formación y desarrollar programas de capacitación; - Fomentar la participación en actividades de desarrollo profesional.

## Discusión

El desarrollo de una matriz de competencias adaptada de la teoría de Kolb^5^ representa un enfoque innovador y robusto para la enseñanza en salud, particularmente orientado al manejo de las condiciones crónicas no transmisibles (CCNT). El ciclo experiencial, que comprende las fases de experiencia concreta, observación reflexiva, conceptualización abstracta y experimentación activa, ofrece una metodología altamente aplicable a los contextos clínicos complejos y desafiantes. Al seguir ese ciclo, los profesionales de la salud no solo adquieren conocimiento teórico, sino que también perfeccionan sus habilidades prácticas mediante la reflexión crítica y la experimentación activa en situaciones reales^6^. Esta integración fomenta el desarrollo de competencias indispensables para el cuidado de condiciones crónicas, que exigen abordajes adaptativos y multidisciplinarios^9^.

La Experiencia Concreta, primera fase del ciclo de Kolb, se alinea con el Eje 1: Relaciones y Comunicaciones Interpersonales e Interprofesionales. En esta etapa, el foco está en la identificación de problemas, el reconocimiento de brechas y la caracterización de las necesidades específicas del cuidado en salud para personas con condiciones crónicas. Este proceso exige la observación activa de situaciones reales y la interacción continua con otros profesionales y con los propios pacientes, lo que es fundamental para el trabajo en equipo eficaz y la comunicación interpersonal. La literatura corrobora la eficacia de la comunicación como un elemento importante en todas las profesiones de la salud y en las instituciones. Ella impulsa la innovación, el desarrollo de estrategias de comunicación y el uso de tecnologías que promuevan una interacción clara y objetiva^10^ ^-^ ^11^. Además, la capacidad de adaptar el lenguaje para diferentes públicos, ya sea el equipo multiprofesional o los pacientes y sus familias, contribuye significativamente a aumentar la adherencia al tratamiento y la percepción de cuidado^11^. Esto ocurre porque una comunicación eficaz aclara dudas, atenúa sentimientos frente a las condiciones clínicas y favorece el establecimiento de un vínculo afectivo, generando beneficios directos para la calidad de la atención y para la recuperación de los pacientes^12^.

Esta fase inicial de la Experiencia Concreta es fundamental, pues establece la base para el compromiso interpersonal e interprofesional, promoviendo la colaboración entre diferentes especialidades. La Atención Primaria Interprofesional (*Interprofessional Primary Care* - IPC), como un modelo de atención colaborativa al paciente, se vuelve cada vez más vital en el escenario de sistemas de salud complejos y ante las crecientes necesidades de los pacientes^13^. De esta forma, la Experiencia Concreta facilita las interacciones colaborativas y se considera el punto de partida para la creación de una matriz de competencias centrada en la atención a las Condiciones Crónicas No Transmisibles (CCNT).

La Observación Reflexiva, segunda fase del ciclo, está directamente vinculada al Eje 2: Profesionalismo y Ética. En esta etapa, el profesional se dedica a reflexionar críticamente sobre las acciones realizadas, analizando los eventos vivenciados y los resultados obtenidos. Los profesionales que trabajan con pacientes crónicos enfrentan desafíos éticos continuos, como garantizar el respeto a la autonomía de los pacientes y la confidencialidad, al mismo tiempo que promueven la justicia social en el acceso a los tratamientos^14^. Para que la atención alcance un nivel de alta calidad ética, es imprescindible que los principios de respeto a la autonomía del paciente y de justicia sean debidamente considerados^15^. La capacidad de reflexionar sobre estas cuestiones permite que los profesionales de la salud mejoren continuamente sus prácticas, asegurando que sus acciones estén de acuerdo con estándares éticos elevados.

En esta fase de observación reflexiva, el aprendizaje experiencial también promueve el desarrollo de una visión crítica y creativa. Los profesionales necesitan ser capaces de contextualizar situaciones complejas y profundizar la reflexión, un proceso que puede describirse como un aprendizaje creativo, dinámico y transformador, que perfecciona la práctica al promover la autoconciencia y el pensamiento crítico^16^.

De esta forma, el Eje 2, que aborda el profesionalismo y la ética, evidencia cómo la observación reflexiva contribuye significativamente a la formación de profesionales de la salud aptos para actuar con responsabilidad y sensibilidad ante los dilemas éticos, especialmente en contextos de larga duración, como las Condiciones Crónicas No Transmisibles (CCNT).

A continuación, la Conceptualización Abstracta, tercera fase del ciclo, corresponde al Eje 3: Enfoque en la Persona, la Familia y la Comunidad. En esta etapa, los profesionales de la salud analizan los datos recolectados y las observaciones realizadas para construir interpretaciones críticas sobre las condiciones de salud de los pacientes. Aquí, el énfasis recae en la capacidad de los profesionales de asociar las condiciones crónicas con los determinantes sociales, culturales y familiares, además de proveer tratamiento, asesoramiento sobre gestión de la salud e intercambio de información, especialmente para individuos que conviven con enfermedades crónicas^17^ ^-^ ^18^. La conceptualización abstracta implica comparar la experiencia actual con situaciones semejantes, generalizar reglas y principios, y sintetizar ideas a partir de discusiones, formando un núcleo común de conceptos compartidos^19^. Este enfoque permite que los profesionales de la salud adapten sus intervenciones de forma holística, atendiendo a las necesidades físicas, emocionales y sociales del paciente.

Esta fase es especialmente relevante en el manejo de las CCNT, donde la adherencia al tratamiento y el seguimiento regular son fundamentales. Al integrar este análisis crítico con el foco en las familias y comunidades, los profesionales quedan capacitados para ofrecer un cuidado que va más allá del tratamiento médico. Esto promueve un abordaje integral de la salud, que incluye la educación del paciente y el fortalecimiento de redes de apoyo comunitarias.

Por último, la Experimentación Activa, última fase del ciclo, se alinea con el Eje 4: Gestión y Evaluación. En esta etapa, los profesionales de la salud aplican el conocimiento adquirido directamente en la práctica clínica. Ello impulsa la transformación de los procesos de trabajo y la implementación de estrategias basadas en evidencias científicas. El núcleo de la experimentación activa es la acción: representa la aplicación práctica de conocimientos y procesos de pensamiento en nuevas situaciones, siempre con énfasis en la colaboración y el trabajo en equipo^20^.

La educación sobre enfermedades crónicas empodera a las personas, proporcionándoles el conocimiento, la competencia y los recursos necesarios para comprender mejor su condición, tomar decisiones eficaces sobre el tratamiento y adoptar elecciones de estilo de vida saludables. Esa educación generalmente abarca información sobre la naturaleza de las condiciones crónicas, incluidas sus causas y síntomas, opciones de tratamiento y estrategias de gestión. En este contexto, el desarrollo profesional continuo es esencial para garantizar que los profesionales de la salud estén siempre actualizados y preparados para enfrentar los desafíos impuestos por las CCNT^21^.

La matriz de competencias propuesta es, por lo tanto, una herramienta indispensable para alinear los principios teóricos estudiados con las necesidades reales de los profesionales de la salud que actúan en el manejo de condiciones crónicas. Al seguir las fases del ciclo de Kolb, los profesionales están capacitados para desarrollar competencias que no solo responden a las demandas técnicas del cuidado, sino que también promueven la reflexión crítica, el análisis contextual y la experimentación práctica, todos esenciales para el enfrentamiento de los desafíos complejos asociados a las CCNT. 

Adicionalmente, la matriz de competencias propuesta refuerza la necesidad de una Educación Permanente en Salud. Esta necesidad es ampliamente enfatizada por el Plan de Acciones Estratégicas para el Enfrentamiento de las Enfermedades Crónicas y Agravios No Transmisibles (Plano DANT), que preconiza el desarrollo continuo de las habilidades de los profesionales de la salud para lidiar con las crecientes demandas de las Enfermedades Crónicas No Transmisibles (DCNT)^21^. Este perfeccionamiento es esencial no solo para mejorar la calidad de la atención prestada, sino también para garantizar que los profesionales estén aptos para actuar de manera proactiva e innovadora en el complejo contexto de la salud pública.

Así, la matriz de competencias, al integrar la teoría con la práctica por medio del aprendizaje experiencial, promueve un enfoque que prepara a los profesionales de la salud para transformar la realidad del cuidado en salud. Al unir las cuatro fases del ciclo de Kolb con los cuatro ejes de la matriz, los profesionales están preparados para enfrentar las condiciones crónicas de manera innovadora, eficiente y humanizada, contribuyendo a la mejora continua de los servicios de salud y al bienestar de las comunidades atendidas.

La investigación señala que la mayoría de los egresados estaba compuesta por enfermeros. De este modo, la matriz de competencias refuerza la importancia del enfermero como uno de los pilares para la cooperación en la atención. Se reconoce la habilidad y el conocimiento de estos profesionales para orientar actividades educativas junto a pacientes, familia, comunidad y equipos, conectándolos en redes de apoyo. La práctica se ajusta a la realidad local, posibilitando al enfermero ofrecer un cuidado más eficiente, humanizado y centrado en las verdaderas necesidades de las personas. Ello contribuye de forma significativa a la mejora de los resultados de salud y a la efectuación de la atención integral.

Este estudio, al basarse exclusivamente en datos cualitativos provenientes de proyectos de intervención, presenta limitaciones inherentes a su diseño metodológico. Este enfoque puede restringir la amplitud de las evidencias sobre la eficacia de la matriz de competencias en diferentes contextos de práctica clínica, dificultando la generalización de los hallazgos. Además, por tratarse de un análisis documental *ex post facto*, no fue posible realizar entrevistas o triangulaciones complementarias para profundizar los resultados.

Para investigaciones futuras, se recomienda la realización de estudios longitudinales que puedan evaluar la aplicación y el impacto de la matriz en diversos escenarios de salud y regiones. La inclusión de metodologías cuantitativas también resulta relevante para medir de forma más robusta los resultados obtenidos con el uso de la matriz de competencias. Otra oportunidad sería el desarrollo de estudios comparativos entre diferentes enfoques educacionales, a fin de verificar el potencial de replicabilidad del aprendizaje experiencial en otros contextos de salud y formación profesional. Tales investigaciones podrían ampliar la validez externa de la propuesta y proporcionar una comprensión más amplia de su impacto.

## Conclusión

El análisis de los proyectos de intervención, realizado por medio del modelo de aprendizaje experiencial de Kolb, permitió revelar un conjunto de competencias que apoyan la gestión de la práctica clínica en el ámbito de las Condiciones Crónicas de Salud (CCS). Este enfoque no solo combina la teoría con la práctica para proporcionar una formación que prepare a los profesionales de la salud para enfrentar los desafíos de las CCS en los más variados contextos, sino que también amplía el alcance de actuación de la Enfermería como campo protagonista en la promoción de la salud.

La matriz de competencias desarrollada, además de preparar especialistas para actuar con CCS, refuerza la importancia del pensamiento crítico, de las características analíticas y de la aplicación del conocimiento, promoviendo una mayor comprensión de cuestiones complejas en el aprendizaje en salud.

## Data Availability

Todos los datos generados o analizados durante este estudio están incluidos en este artículo publicado.
